# 1231. Microbial Profile of Biliary Stent, Bile and Surgical Site Cultures in Pancreaticoduodenectomy Patients – Implications for Surgical Prophylaxis

**DOI:** 10.1093/ofid/ofac492.1063

**Published:** 2022-12-15

**Authors:** Kai Chee Hung, Winnie Lee, Andrea Layhoon Kwa, Ye Xin Koh, Brian Kim Poh Goh, Jasmine Chung

**Affiliations:** Singapore General Hospital, Singapore, Singapore; Singapore General Hospital, Singapore, Singapore; Singapore General Hospital, Singapore, Singapore; Singapore General Hospital, Singapore, Singapore; Singapore General Hospital, Singapore, Singapore; Singapore General Hospital, Singapore, Singapore

## Abstract

**Background:**

Surgical site infection (SSI) rates post pancreaticoduodenectomy (PD) range from 20 to 40%. Biliary obstruction (± stent in situ) leads to colonization of the biliary tract with gut flora, and bile contamination during surgery increases SSI risk. Current guidelines recommend surgical prophylaxis (ppx) with 1^st^ or 2^nd^ generation cephalosporins. However, emerging data favor targeted ppx based on biliary cultures to reduce SSI. We reviewed the microbial profile of pathogens isolated from perioperative biliary stent and bile cultures, and evaluated the impact of ppx on culture positive SSIs within 30 days of PD.

**Methods:**

This was a retrospective study conducted in Singapore General Hospital. Patients who had PD from 1/1/2013 to 31/12/2019 were included. The first positive cultures from perioperative biliary stent, bile, and SSI cultures obtained within 30 days of surgery were reviewed. Antibiotic use 30 days prior and during surgery was collected.

**Results:**

A total of 341 patients with a mean age of 63.9 ± 11.3 years were included; 202 (59.2%) were male and 137 (40.2%) received antibiotics in the past 30 days. For surgical ppx, patients received either cephalosporins ± metronidazole (216 [63.3%] ceftriaxone, 25 [7.3%] cefazolin), piperacillin-tazobactam (TZP) (35 [10.3%)], ciprofloxacin ± metronidazole (16 [4.7%)] or other antibiotics (14 [4.1%]), while 35 had missing data. Of the positive cultures from 84 biliary stent, 86 bile and 79 SSI, the most common organisms isolated were *Enteroccocus* spp., *E. coli*, *Klebsiella* spp., *Enterobacter* spp. and *Candida* spp. Ceftriaxone susceptibility rates in *E. coli* were 50.0%, 54.8% and 22.2% in biliary stent, bile and SSI cultures respectively; similarly low rates were also seen in *Klebsiella* spp. (Tables 1-3). Antibiotic use 30-days prior was associated with higher ceftriaxone resistance rates in *E. coli* and *Klebsiella* spp. from bile cultures (70.8% vs 36.4%, p=0.019). In patients with biliary stents cultured, use of TZP ppx showed a trend towards lower rates of culture positive SSIs compared to non-TZP ppx (9.1% vs 29.4%, p=0.054).

**Figure:**
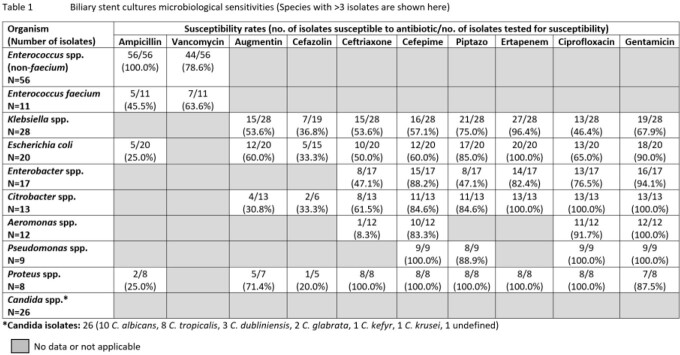

**Figure:**
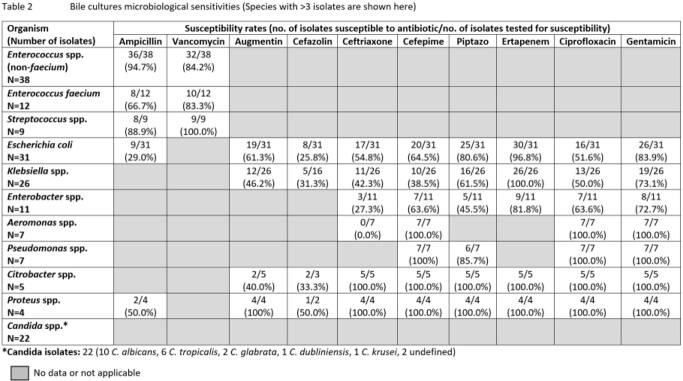

**Figure:**
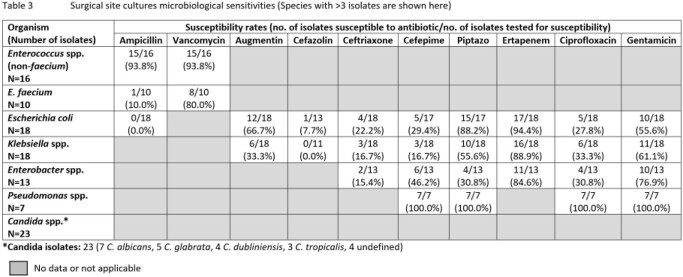

**Conclusion:**

Our local gram-negative bacteria susceptibility rates to ceftriaxone are low in biliary stent, bile fluid cultures, and even lower for SSI post PD. Antibiotic ppx for PD in high-risk patients may need to be broadened.

**Disclosures:**

**All Authors**: No reported disclosures.

